# Mental health literacy and associated factors among traditional healers of Jimma town, southwest, Ethiopia 2020: a community based, cross-sectional study

**DOI:** 10.3389/fpsyt.2024.1304454

**Published:** 2024-05-22

**Authors:** Gudeta Mideksa, Elias Tesfaye, Yimenu Yitayih, Yohanes Sime, Kemal Aliye, Abraham Tamirat Gizaw

**Affiliations:** ^1^ Psychiatry Department, Faculty of Medical Sciences, Jimma Medical Center, Jimma, Ethiopia; ^2^ Psychiatry Department, Faculty of Medical Sciences, Jimma University, Jimma, Ethiopia; ^3^ Psychiatry Department, College of Medicine and Health Science, Dilla University, Dilla, Ethiopia; ^4^ College of Health and Medical Science, Department of Psychiatry, Haramaya University, Harar, Ethiopia; ^5^ Department of Health, Behavior and Society, Faculty of Medical sciences, Jimma University, Jimma, Ethiopia

**Keywords:** mental health literacy, traditional healer, mental illness, South West, Ethiopia

## Abstract

**Background:**

Traditional healers are in the front line to give the mental healthcare service in developing countries like Ethiopia. In Ethiopia, different studies were done focusing on the role of traditional medicine and perception of the community toward traditional medicine. However, there is paucity of studies, which shows the level of mental health literacy among traditional healers. Therefore, this study intended to mental health literacy level of traditional healers in Jimma town, Ethiopia.

**Method:**

A community-based cross-sectional study design was employed among 310 participants. To select the participants from Jimma town, a stratified random sampling method was utilized from August 1 to September 30, 2020. The Mental Health Literacy Questionnaire was used to assess mental health literacy for assessment of mental health literacy among traditional healers. The collected data were coded and entered into EpiData version 4.6 and exported to SPSS version 25.0 for analysis. Bivariate and multivariable linear regression was used for data analysis.

**Result:**

The finding of this study showed that the samples of traditional healers found in Jimma town scored a total mean of 95.91 ± 3.0025 for mental health literacy. Age [β = −0.052 (95% CI: −0.078, −0.026)], year of experience [β = 0.095 (95% CI: 0.067, 0.123)], family history of mental illness [β = 1.709 (95% CI: 0.543, 2.360)], history of professional help seeking on mental illness [β = 0.501 (95% CI: 0.715, 2.288)], history of getting information of mental illness on media [β = 0.941 (95% CI: 0.345, 1.538)], training on mental health [β = 2.213 (995% CI: 1.520, 2.906)], history of treating mental illness [β = 1.676 (95% CI: 0.808, 2.544)], and informal education [β = −1.664 (95% CI: −2.081, −1.247)] were factors significantly associated with MHL.

**Conclusion:**

The mental health literacy of traditional healers mean score is lower than the mean score of other studies. Age, year of experience, training on mental illness, family history, history of professional help seeking, history of treating mental illness, information on mental illness, and informal education are significantly associated with mental health literacy. Therefore, structured training is very important to improve their level of mental health literacy.

## Introduction

Health literacy is defined as the extent to which a person is able to make judgment and decision about his/her health condition by obtaining, communicating, and understanding basic health information and service (1). The idea of health literacy helped to construct mental health literacy, which is defined by Australian scholars as “knowledge and beliefs about mental disorders which aid their recognition, management or prevention” (2). Mental health literacy (MHL) encompasses the ability to identify or recognize mental disorders and knowledge of causes, prevention, and treatments of mental illness, which includes first aid skills to support people with mental health problems (3). Mental health literacy enables to understand one’s mental health condition, which helps to take any action based on the information (4). Also, it increases one’s resilience and control over mental health that enhances help-seeking behavior (5).

A traditional healer is an educated or layperson who claims the ability or power of healing to cure ailments. He could have a particular skill to treat specific types of complaints or afflictions and might have gained a reputation in her/his community or elsewhere (6). In many countries, traditional healers are in the front line to give the mental healthcare service especially in countries with insufficient modern mental healthcare facilities, and help-seeking behavior of the community is low (7). In such conditions, the level of mental health literacy of the traditional healers significantly puts negative impact on the quality and effectiveness of the service delivery system of mental illness (8).

In Ethiopia, around 80% of the population uses traditional healing services for mental and other health-related problems (9). The acceptance and the role of the healers in the community are very high, which is related to different factors, but commonly like most of the African countries, it is related to the inaccessibility of modern psychiatric treatment and cultural or religious beliefs of mental illness (10).

Most studies indicated that the level of mental health literacy and healing practice of traditional healers relies on the indigenous knowledge and culture of their community (11). Western traditional healers have a better level of understanding and recognition of mental illness that they more related mental illness with the bio-psychosocial factors (12). However, the level of understanding of mental illness among traditional healers in sub-Saharan African countries is low and most of the studies recommended giving awareness and training on mental illness to raise their understanding (13).

Low mental health literacy of traditional healers puts its negative impact on mental illness service delivery by delaying formal treatment access, which directly affects the prognosis and outcome of the illness (14). Also, the low awareness of mental illness leads the healers to provide unsafe and harmful full remedies, which result in exacerbation of the condition and cause other additional adverse health effects (15).

According to different studies, mental health literacy on a traditional healers level is affected by different factors such as age, sex, income, marital status, educational status, and occupational status, history of treating mentally ill, training on mental illness, types of training on traditional healing, family history of mental illness, social support, information on mental illness, and year of experience on traditional healing (16).

Although formal coordination and continuous activities were not observed previously in the promotion of mental health conditions in the community in general, some efforts were attempted by governmental and non-governmental institutions to create awareness through different campaigns and programs especially using electronic media (17). In a Lancet Commission report on global mental health and sustainable development (18), the authors emphasized the significance of recognizing traditional healing practices within the broader framework of mental healthcare. The report outlined the importance of addressing the knowledge and attitudes of traditional healers to promote effective collaboration between traditional healing and modern psychiatric services.

A study by Gureje et al. (19) emphasized the importance of acknowledging and integrating traditional and complementary systems of medicine, including traditional healing practices, into mental healthcare. The study highlighted the need to understand the specific knowledge and beliefs of traditional healers regarding mental health issues (19).

Tesfaye et al. (20) provided insights into the challenges and opportunities of developing mental healthcare plans in low-resource settings, emphasizing the need to engage traditional healers as key stakeholders in mental health literacy and service delivery (20). Teferra and Shibre (21) conducted a qualitative study focusing on the perceived causes of severe mental disturbance and preferred interventions by the Borana semi-nomadic population in southern Ethiopia. The study shed light on the cultural and social factors influencing the mental health literacy of traditional healers within specific communities (21).

Because of the increasing interest in traditional means of mental illness treatment globally, including Ethiopia, different studies were done focusing on the role of traditional medicine and the perception of the community toward traditional medicine (9, 22, 23). Despite the growing body of literature on mental health literacy and traditional healing practices, there remains a research gap in understanding the specific knowledge, attitudes, and practices related to mental health among traditional healers in Jimma town. Few studies have explored the unique cultural and social factors that influence the mental health literacy of traditional healers in this setting. Despite the important role that traditional healers play in providing healthcare services in Ethiopia, there is little knowledge and research on their level of mental health awareness. Traditional healers are frequently the first point of contact for individuals seeking help for mental health issues in Ethiopia. However, there is a lack of knowledge and awareness about common mental health conditions, appropriate treatment approaches, and potential risks associated with traditional healing practices. This lack of mental health literacy among traditional healers may result in misdiagnosis, ineffective treatment, and further stigmatization of people suffering from mental illnesses. As a result, investigating traditional healers’ mental health literacy in Ethiopia is critical for developing effective strategies to improve their capacity to support mental health services and promote better outcomes for people facing mental health issues.

Additionally, addressing this research gap is essential for promoting holistic and integrated mental healthcare that values traditional healing practices alongside modern psychiatric services. Thus, the finding of this study may provide vital information for the community, health sectors, and policymakers regarding the level of mental health literacy of traditional healers in Jimma town. Furthermore, for the participants it may create insight toward their level of recognition, knowledge, and attitude toward mental health in general. Finally, since limited research has been done in Ethiopia concerning this issue, it may be used as an input for further research.

## Method and materials

### Study design

A community-based cross-sectional study design was utilized.

### Study area and period

The study was conducted in Jimma town, Southwest Ethiopia, from August 1 to September 30, 2020. Jimma town is located 352 km southwest of Addis Ababa, the capital city of Ethiopia. The town covers an area of 167 ha, and it has a latitude and longitude of 7°40′N, 36°50′E. The town has 17 administrative kebeles, and the number of households reported in the town was approximately 32,191. The town’s total population from the 2007 Central Statistical Agency (CSA) census was reported to be 120,960 (24). The town has one medical center and one general hospital. Psychiatric outpatient and inpatient services are available only at the medical center. There are a total of 780 traditional healers in Jimma town.

### Source of population

All traditional healers were found in Jimma town.

### Study population

Traditional healers were found in Jimma town during the study period.

### Eligibility criteria

Traditional healers living in Jimma town and presented during data collection were included. However, traditional healers who were acutely ill during the data collection period and unable to communicate were excluded from the study.

### Sample size determination

The sample size for this study was calculated using a formula for a single population means with the following assumptions.

Where

▪ Margin of error 5% (d = 0.05)
▪ n = minimum required sample size
▪ Zα/2= Z value at (∞ = 0.05) = 1.96
▪ n_f_ = finite population sample size
▪ N = total population

For this study, standard deviation (SD = 10.83) and sampling error (SE = 1) were used from the study conducted in the United States because there is no study so far in Ethiopia and Africa (25).


n=(Zα2×σ)2E2=1.962×10.83212=451

Since the total populations of traditional healers are less than 10,000 in Jimma town, the finite population correction formula was used.


$${\text{n}}_{\text{f}}=\frac{n}{1+\frac{n}{N}}=\frac{451}{1+\frac{451}{780}}=\frac{451}{1.56}=290$$

By adding 10% of non-response rate, the final sample size was 310. If a participant initially does not respond to the data collection efforts, researchers often make multiple attempts to reach out to them. This may involve sending reminder emails, making phone calls, or scheduling additional visits to their location to collect the data.

### Sampling procedure

The participants in this study were chosen using a stratified random sampling technique. Jimma town had 780 traditional healers, who were divided into four strata based on the foundation of their healing practice. The total number of traditional healers in each stratum was 375 for Muslims, 340 for Orthodox Tewahedo Christians, 50 for Protestant Christians, and 15 for herbalists. Information was obtained from each traditional healer’s focal person. Following classification, the calculated sample size was proportionally allocated to each stratum, with 154 for Muslim traditional healers, 139 for Orthodox Tewahedo, 21 for Protestant, and six for herbalist traditional healers. Finally, after receiving a complete list of traditional healers from the corresponding offices in each stratum, the study units were chosen by lottery from each stratum.

The Mental Health Literacy Scale (MHLQ) was used for the assessment of mental health literacy. The MHLQ scale contains 35 items and the first 15 items (1 to 15) rated on a 4-point Likert scale (1—very unlikely/unhelpful to 4—very likely/helpful) and the rest items (16 to 35) rated on a 5-point Likert scale (1—strongly disagree/definitely unwilling to 5—strongly agree/definitely willing). The total score is produced by summing all items, the maximum and minimum scores are 160 and 35, respectively, and the highest score is indicated higher mental health literacy. The internal consistency, Cronbach’s alpha coefficient of MHLQ in the current study was 0.763. According to an Australian study, the total reliability of Cronbach a was 0.79 (26).

Level of social support: For this study, social support is measured using the Oslo three-item social support scale and a score of −3–8 is poor support, 9–11 is moderate support, and 12–14 is strong support (27). Mental health experience and traditional healing factors were assessed using five- and two-item questionnaires, respectively. The data were collected by face-to-face interviews by pretested interviewer-administered questionnaires. Four data collectors (BSc psychiatric professionals) were employed for 1-month data collection periods and supervised by one supervisor. Then, the data were collected from available traditional healers in Jimma town.

If a participant initially does not respond to the data collection efforts, data collectors often make multiple attempts to reach out to them. This may involve making phone calls or scheduling additional visits to their location to collect the data.

The questionnaire was prepared first in English and translated into Afan Oromo and Amharic language then back translated to English to check the consistency by a professional. The training was given for data collectors and supervisors. The pretest was conducted on 10% (28) of the participants at Agaro town to identify potential problems in data collection tools and modification of the questionnaire and the MHLQ scale reliability was checked, and the reliability of Cronbach’s alpha is 0.76. Regular supervision and support were given for data collectors by the supervisor and principal investigator. Data were checked for completeness and consistency by supervisors and principal investigators daily during data collection time.

## Operational definition

**Mental health literacy:** The 35-item MHLQ scale was used to measure the level of MHL, which has a score range from 35 to 160, and the increased score indicates the increased level MHL (26).

**Ability to recognize:** one’s capability to identify manifestations of mental health problems, a specific disorder, or a group of mental disorders.

**The knowledge of how to seek mental health information:** the knowledge of how and where to get access to the information and applying that information accordingly.

**Knowledge of risk factors and causes:** one’s knowledge for different factors likes biological, environmental, or genetically those that increase the risk of developing mental health problems.

**Knowledge of self-treatment:** the knowledge of specific types of treatment suggested by professionals of mental health that someone is able to practice.

**Attitudes that promote recognition and appropriate help-seeking:** attitudes affecting recognition of the disorder and help-seeking willingness.

**Traditional healers:** Faith healers and herbalists.

**Faith healers:** Imams/Sheikh’s of Islam religion, Priests of Orthodox Tewahedo Christian religion and Pastors of Protestant Christian religion.

**Social support:** OSLO 3-item used to measure social support which has a score range from 3 to 14, and the increased score indicates the increased level of social support.

## Data processing, analysis, and presentation

Data were coded and entered into EpiData Version 4.6 and exported to IBM SPSS Statistics Version 25.0 for analysis, and before data analysis, linear assumptions were checked. The normality of data distribution was checked by histogram and objectively by Kolmogorov–Smirnov and Shapiro–Wilk tests, and the data were normally distributed; multicollinearity was checked using VIF and tolerance test, and there was no issue of multicollinearity. The values of tolerance for all variables were greater than 0.2, and all values of VIF were less than 10. Linearity was checked by scatter plot, and the data were linear.

A descriptive statistic was used to present descriptive data. Mean, frequency, and the percentage were used to describe the outcome variable, and the result was presented using charts and tables. Correlation analysis was used to determine the relationship between mental health literacy and independent variables. All variables were entered into bivariable linear regression to identify associated factors of mental health literacy and variables with *p*-value< 0.25 were considered as candidates for multivariate linear regression analysis. In multivariate linear regression analysis, variables with p-value< 0.05 were considered as statistically significant.

## Results

### Description of sociodemographic characteristics of the study participants

A total of 310 traditional healers participated in this study with a response rate of (96.7%). Approximately half (155) of the participants were Muslims by religion, and almost all of the participants (304; 98.1%) were private organization workers. More than half 174 (56.1%) of the participants were Oromo by ethnicity, and the majority of 288 (92.9%) of the participants were married. Regarding the level of education, 130 (41.9%) had informal education and 32 (10.3%) were college level and above. The total mean (± SD) age of the participants was 41.22 + 9.03 years and ranged from 28 to 67 years. The median score of the average monthly income of the participant was 3,000 Ethiopian Birr with an interquartile range of 2,000 Ethiopian Birr (**Table 1**).

**Table 1:** The basic sociodemographic characteristics of traditional healers found in Jimma town, Ethiopia 2020.

Variables	Variable categories	Frequency (n = 351)	Percentage (100)
Types of traditional Healers	Imam/Sheikh	149	48.1
	Orthodox Tewahedo Christian Priest	136	43.9
	Protestant pastor/clergy	19	6.1
	Herbalist	6	1.9
Marital status	Married	288	92.9%
	Single	22	7.1%
Religion	Muslim	155	50%
	Orthodox Christian	136	43.9%
	Protestant Christian	19	1.9%
Ethnicity	Oromo	174	56.1%
	Amhara	132	42.6
	Tigre	4	1.3
Occupational status	Private worker	304	98.1
	Merchant	6	1.9
Educational status	Informal education	130	41.9
	Primary	66	21.3
	Secondary	49	15.8
	Preparatory	33	10.6
	College and above	32	10.3

### Mental health literacy

The finding of this study showed that the samples of traditional healers found in Jimma town scored the total mean ( ± SD) score of 95.91 ± 3.0025 for mental health literacy (MHL), which was measured using the mental health literacy questioner (35-MHLQ).

### Mental health experience of the study participants

Based on the finding of this study, majority of participants, 286 (92.3%), do not have a family history of mental illness and also most of the participants, 285 (91.9%), never seek professional help of mental illness for themselves previously. Most of the participants, 257 (82.9%), did not take any form of training on mental illness. However, 269 (86.8%) of participants previously got information on media about mental illness.

### Traditional healing-related factors

The mean (± SD) year of experience in traditional healing was 13.48 ± 8.45 with a range of 1–37 years of experience. On types of training on traditional healing majority of participants, 297 (95.8) were trained informally and the remaining 13 (4.2%) were trained formally.

### Social support

Regarding the social support of the participants as measured by OSSS-3, out of the 310 healers, the mean (± SD) was 13.83 ± 0.4 with a range of 11–14 on the Oslo Social Support Scale.

### Factors associated with mental health literacy

Bivariate and multivariable regression analyses were done to identify predictors of mental health literacy among traditional healers. Bivariate analysis variables like participants’ age, monthly income, year of experience on traditional healing, types of training on traditional healing, social support, family history of mental illness, history of having information on media about mental illness, having a history of professional help-seeking on mental illness for self or others, training on mental health, history of treating mental illness, and informal and preparatory educational status were found to have a statistical association with the outcome variable and considered as a candidate for the final model (**Table 2**).

**Table 2:** Bivariate linear regressions of MHL of traditional healers.

Variable	Category	R^2^	Unstandardized B	95% CI		p-value
				Lower bound	Upper bound	
Age		.044	−.070	−.106	−.034	.000
Level of education	Informal education	.257	−3.077	−3.664	−2.490	.000
	Primary	.001	.230	−.590	1.050	.581
	Secondary	.003	.471	−.448	1.390	.314
	Preparatory	.024	1.492	.415	2.568	.007
	College and above^*^					
Marital status	Single	.001	.440	−.867	1.747	.508
	Married^*^					
Occupation	Merchant	.000	.402	−2.005	2.869	.727
	Private organization^*^					
Monthly income		.309	.001	.001	.002	.000
Types of training on traditional healing	Formal	.026	2.423	4.076	.769	.004
	Informal^*^					
Year of experience on traditional healing		.171	.146	.110	.182	.000
Family history of mental illness	Yes	.310	−6.018	5.012	7.024	000
	No^*^					.
History of having information on media about mental illness	Yes	.161	3.550	2.642	4.458	000
	No^*^					.
History of professional help seeking on mental illness	Yes	.274	5.754	4.703	6.806	.000
	No^*^					
Training on mental illness	Yes	.428	5.207	4.532	5.881	.000
	No^*^					
History of treating mental illness	Yes	.172	5.174	3.900	6.448	000
	No^*^					.
Social support		.067	1.920	1.117	2.723	.000

*Reference.

In the final multivariable linear regression analysis, participants’ age [β: −0.052, 95% CI 0.078–(−0.026)], year of experience on traditional healing [β: 0.095, 95%, CI 0.067–0.123], family history of mental illness [β: 1.452, 95% CI 0.543–2.360], history of professional help seeking on mental health for self or others [1.501, 95% CI 0.751–2.288], history of having information on media about mental illness [β:.941, 95% CI 0.345–1.538], training on mental health [β: 2.213, 95% CI 1.520–2.906], history of treating mental illness [1.676, 95% CI.808–2.544], and informal education [β: −1.664, 95% CI 1.247–2.081] were the variables that significantly associated with level of MHL, and these variables contribute 75.2% of the variation in the mental health literacy of traditional healers (R = 0. 867, R^2 =^ 0. 752, F = 75.176, p< 0.001) (**Table 3**).

**Table 3:** Multivariable linear regression model for MHL of traditional healers.

Variables	Category	Unstandardized coefficients	Sig.	95.0% confidence interval for B		Collinearity statistics	
		B		Lower bound	Upper bound	Tolerance	VIF
Constant		88.87	.000	82.506	95.245		
**Age**		−0.052	<0.001*	−0.078	−0.026	0.527	1.898
**Educational status**	College and above	1	1	1	1	1	1
	Informal education	−1.664	<0.001*	−2.081	−1.247	0.684	1.462
	Preparatory	.234	0.449	−0.374	0.842	0.824	1.213
**Year of experience**		0.095	<0.001*	0.067	0.123	0.516	1.939
**History of professional help seeking on mental illness**	Yes	1.501	<0.001*	0.715	2.288	0.632	1.582
	No	1	1	1	1	1	1
**History of getting information on media about mental health**	Yes	0.941	0.002*	0.345	1.538	0.710	1.409
	No	1	1	1	1	1	1
**Training on mental health**	Yes	2.213	<0.001*	1.520	2.906	0.425	2.351
	No	1	1	1	1	1	1
**History of treating mental illness**	Yes	1.676	<0.001*	0.808	2.544	0.668	1.497
	No	1	1	1	1	1	1
**Family history of mental illness**	Yes	1.452	0.002	0.543	2.360	0.457	2.187
	No	1	1	1	1	1	1

1 Reference, * significant association.

## Discussion

This study aimed to determine the level of mental health literacy among traditional healers, and the mean of the present study was 94.46 ± 3. Age, level of education, year of experience on traditional healing, types of training on traditional healing, family history of mental illness, information about mental illness on media, seeking professional help on mental health for self or others, training on mental health, and willingness to work with mental health professionals on mental illness are the main predictors on mental health literacy among traditional healers.

The present finding of the MHL mean score is lower than the MHL mean of protestant clergies (134.20 ± 10.83) found in the USA. The discrepancy might be due to the better access to mental health sources of information and better level of education in the United States, which helps them to use modern means of media sources for gathering information. Additionally, the difference might be related to the strong coordination between the clergies and health professionals that create the opportunity to share mental health information (25).

Similarly, the mean of the current finding with the study done in Australia among the community (127.38±) and mental health professionals (145.49±) is lower than in both groups. The discrepancy might be related to accessibility of mental health information in Australia, and the difference with mental health professionals might be due to the formal training they have on the area of mental health (29).

Another study on MHL was done among the Iranian community, and the MHL mean score was 102.75 ± 10.17, which is slightly higher than the current study. The difference might be due to better access for mental health information among the Iranian community (30).

Informal education significantly and negatively influenced the MHL level (β: −1.664, p = .000). This means that participants having informal education more likely have 1.664 times lower MHL level than those who have college and above educational status. This association could be related to the decreased understanding of the scientific explanation of mental health-related conditions and limited knowledge of using modern technologies as resources of information among informal educational status individuals. This finding also agrees with other studies done in Bangladesh, Malaysia, and China (28, 31, 32).

The present study found a negative and significant association between MHL and age (β: −0.052, p< 0.001). This means that a unit increase in age leads to an average of 0.052 unit decrease in the level of MHL. The reason may be related to the different social-political environments that different generations have experienced. This finding is in line with studies done in China and South Korea (28, 33).

Having a family history of mental illness positively and significantly influenced the MHL level (β: 1.452, p = .002), and this association was interpreted as participants who have a family history of MI more likely have 1.452 times higher level of MHL compared with those who do not have. The possible explanation for this finding is individuals with a family history of mental disorders who have the chance to share the experience that could increase the chance to be more familiar with the manifestations and specific mental disorders that contribute to the increase of the MHL level. This finding also agrees with studies done in Iran and Gimbi (30, 34).

The present study found a positive and significant association between MHL and year of experience in traditional healing (β: 0.0950, p = .000). This means that a unit increase in the year of experience on traditional healing leads to an average of 0.0950 unit increase in the level of MHL. The possible explanation for this finding is that working on traditional healing for a longer duration creates the chance to be familiar, to recognize, and to understand different mental illness manifestations, which help to change one’s attitude and knowledge. The finding is in line with the USA and Nigerian study (35, 36).

The present study found a positive and significant association between MHL and having information about mental illness on media (β:.941, p = .002). This means that participants having information about mental illness on media more likely have 0.941 times higher level of MHL than those who do not have. This is because of the capacity of information on shaping individual knowledge and attitude toward mental illness, which finally determines the level of MHL. This result is in line with the studies done in Zimbabwe, A. A, and Gimbi (34, 37, 38).

The present study found a positive and significant association between MHL and training on mental health (β: 2.213, p = .000). The interpretation is that participants who have training on mental health more likely have 2.213 times higher levels of MHL than those who do not have. This significant association could be related to the direct and strong effect of mental health training on enhancing one’s knowledge of mental disorders. The finding of our study is supported by the study done in the USA and Zimbabwe (25, 37).

The present study found a positive and significant association between MHL and having a history of treating mental illness (β: 1.676, p = .000). The interpretation is participants who have a history of treating mental illness more likely have 1.676 times higher levels of MHL than those who do not have. The possible explanation for this finding is that healing mental illness creates an opportunity to be more exposed to a different mental health condition, which helps to have an awareness of mental health. This finding is consistent with the study done in USA (35).

The present study found a positive and significant association between MHL and having a history of professional help-seeking on mental illness for self/others (β: 1.501, p = .000). The interpretation is that participants who have a history of professional help-seeking more likely have a 1.676 times higher level of MHL than those who do not have. This association might be related to the increased probability of getting information on mental health while visiting the professionals during treatment for self or others, which directly affects one’s MHL level. This finding is consistent with the study conducted in A.A (38).

This study attempts to measure the level of mental health literacy of traditional healers of Jimma town; however, while conducting this research, there were different limitations the researcher faced such as the following: Since the research design was cross-sectional, causality cannot be confirmed. Thus, in this study, the relationship between MHL and independent factors cannot be considered as cause and effect. Response bias may be introduced related to some measurement tools we used in this study that required recall of past history. The deficit of literatures on this topic forced the researcher to compare and contrast the finding of our study population with different population groups.

## Conclusion

The MHLQ scale of traditional healers has a lower mean score than other studies. Age, years of experience, mental illness training, family history, history of seeking professional help, history of treating mental illness, mental illness information, and informal education are all significantly associated with MHL. Therefore, Training and Education Programs create and implement customized training programs that emphasize mental health awareness, recognition of common mental health conditions, and knowledge of appropriate referral pathways. These programs must be culturally sensitive and delivered in collaboration with mental health professionals to ensure.

Research initiatives must be supported to understand the impact of MHL interventions on traditional healers’ practices and the well-being of the communities they serve. Engaging traditional healers in research activities can also foster a sense of empowerment and collaboration. By implementing these specific recommendations, efforts can be made to enhance the mental health literacy of traditional healers, leading to improved mental health support within their communities while respecting and complementing traditional healing practices.

For practice, culturally sensitive mental health services should be promoted by working with traditional healers to create culturally sensitive mental health services that combine traditional healing practices with evidence-based treatments. For training and capacity building, initiatives should be implemented to improve traditional healers’ knowledge and skills in mental health literacy and effective referral pathways.

For education, training programs should be developed by creating educational programs on mental health literacy for traditional healers, focusing on topics such as recognizing common mental health disorders, understanding evidence-based treatments, and promoting mental illness destigmatization. Cross-cultural communication should be promoted by teaching cross-cultural communication and collaboration skills to help traditional healers and mental health professionals form effective partnerships.

## Data availability statement

The raw data supporting the conclusions of this article are not publicly available due to participant confidentiality. Data will be made available upon reasonable request from the corresponding author.

## Ethics statement

The studies involving humans were approved by institutional review board of Jimma University. The studies were conducted in accordance with the local legislation and institutional requirements. The participants provided their written informed consent to participate in this study.
