# Corrigendum: Mental health literacy and associated factors among traditional healers of Jimma town, southwest, Ethiopia 2020: a community based, cross-sectional study

**DOI:** 10.3389/fpsyt.2025.1563989

**Published:** 2025-02-11

**Authors:** Gudeta Mideksa, Elias Tesfaye, Yimenu Yitayih, Yohanes Sime, Kemal Aliye, Abraham Tamirat Gizaw

**Affiliations:** ^1^ Psychiatry Department, Faculty of Medical Sciences, Jimma Medical Center, Jimma, Ethiopia; ^2^ Psychiatry Department, Faculty of Medical Sciences, Jimma University, Jimma, Ethiopia; ^3^ Psychiatry Department, College of Medicine and Health Science, Dilla University, Dilla, Ethiopia; ^4^ College of Health and Medical Science, Department of Psychiatry, Haramaya University, Harar, Ethiopia; ^5^ Department of Health, Behavior and Society, Faculty of Medical sciences, Jimma University, Jimma, Ethiopia

**Keywords:** mental health literacy, traditional healer, mental illness, South West, Ethiopia

In the published article, there was an error in affiliation 4. Instead of “Institute of Health, Faculty of Public Health, Department of Health, Behavior and Society, Jimma University, Jimma, Ethiopia”, it should be “College of Health and Medical Science, Department of Psychiatry, Haramaya University, Harar, Ethiopia”.

The authors apologize for this error and state that this does not change the scientific conclusions of the article in any way. The original article has been updated.

